# Supplementary material for: Crystal Structure of the Heterotrimeric Integrin-Binding Region of Laminin-111
Source: Structure. 2017 Mar 7;25(3):530–5. doi: 10.1016/j.str.2017.01.002 (PMC5343747; doi:10.1016/j.str.2017.01.002)
Supplement: Document S1. Figures S1 and S2 [file mmc1.pdf]

Structure, Volume *25*

Supplemental Information

Crystal Structure of the Heterotrimeric Integrin-Binding Region of Laminin-111

David Pulido, Sadaf-Ahmahni Hussain, and Erhard Hohenester

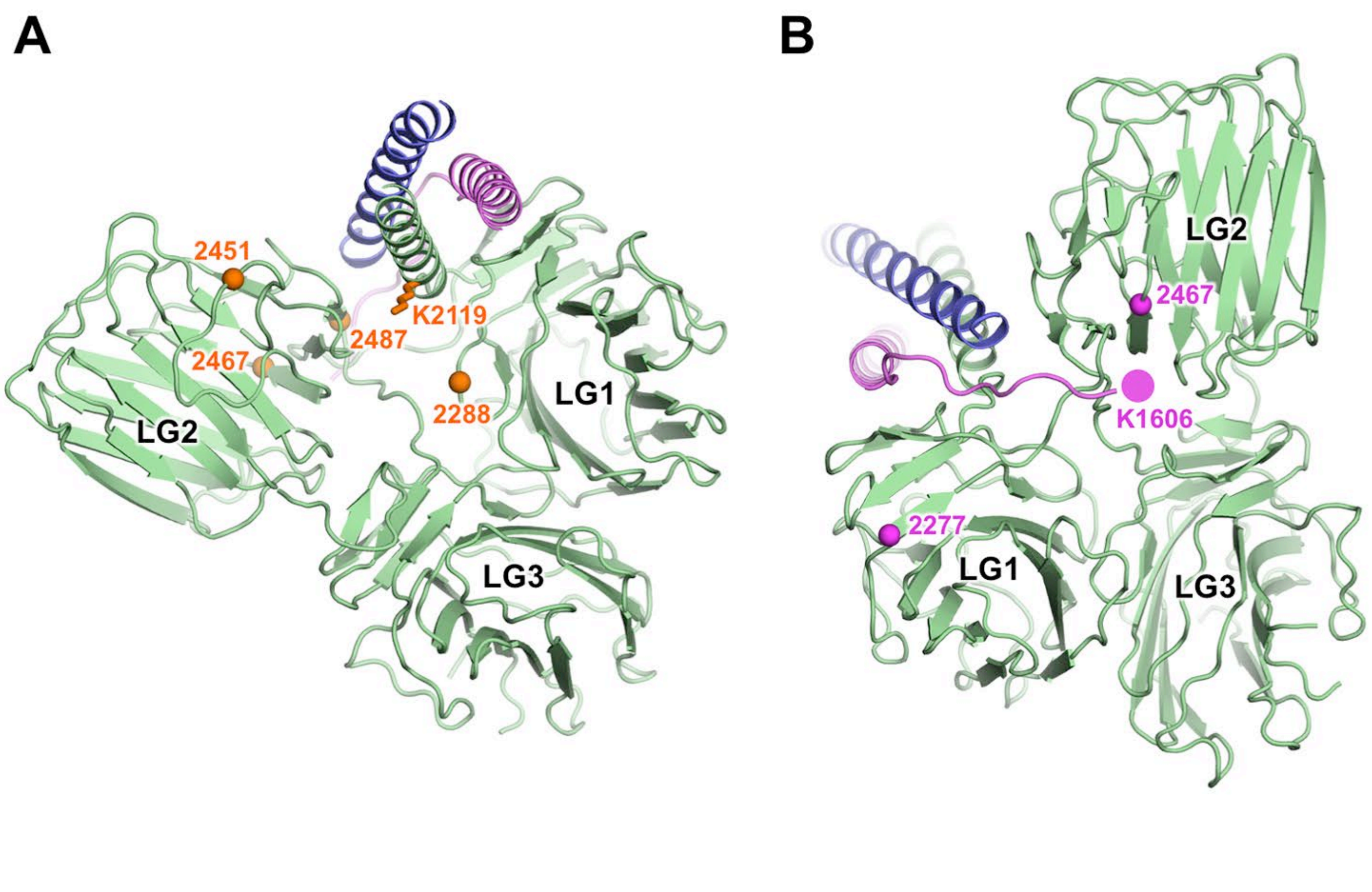

Figure S1, related to Figure 1. Mapping of chemical cross-links onto the mini-E8 structure. The cross-link data are from Armony et al. (2016). (**A**) Cross-links within the laminin α1 chain. α1 Lys2119 (orange sticks) forms cross-links with α1 Lys2288, α1 Lys2451, α1 Lys2467 and α1 Lys2486 (orange Cα spheres). (**B**) Cross-links between the laminin γ1 tail and the α1 chain. γ1 Lys1606 (magenta filled circle adjacent to the last resolved residue of γ1 chain, Glu1605) forms cross-links with α1 Lys2277 and α1 Lys2467 (magenta spheres).

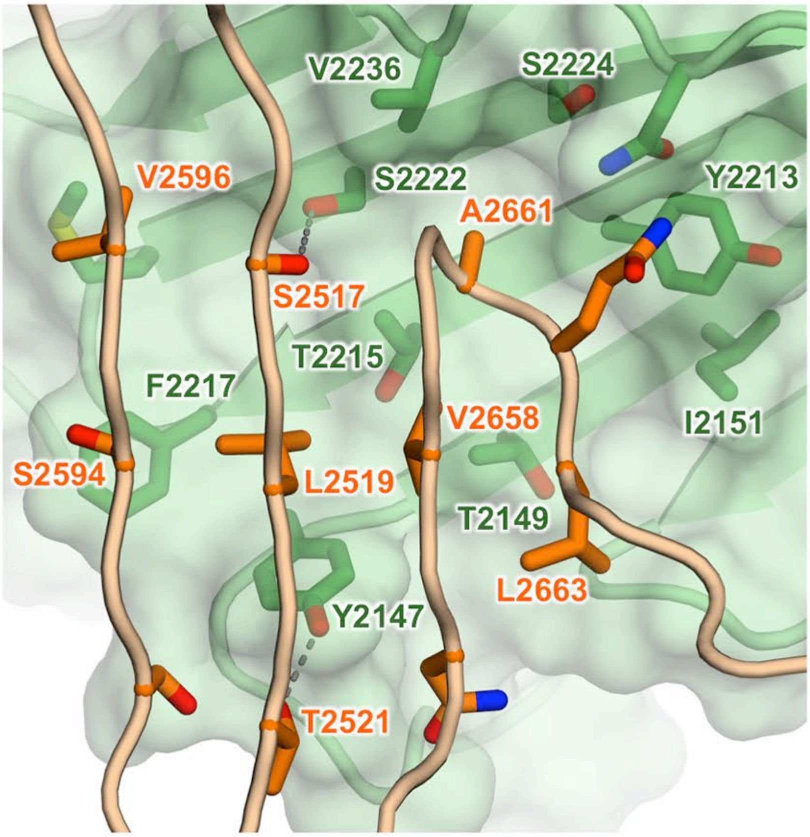

Figure S2, related to Figure 3. The LG1-LG3 interface in laminin-111 mini-E8. LG1 is shown as a transparent green surface, and LG3 is shown in orange.
