# Supplementary material for: Crystal Structure of the Heterotrimeric Integrin-Binding Region of Laminin-111
Source: Structure. 2017 Mar 7;25(3):530–5. doi: 10.1016/j.str.2017.01.002 (PMC5343747; doi:10.1016/j.str.2017.01.002)
Supplement: Document S2. Article plus Supplemental Information [file mmc2.pdf]

Structure

Short Article

Crystal Structure of the Heterotrimeric Integrin-Binding Region of Laminin-111

Graphical Abstract

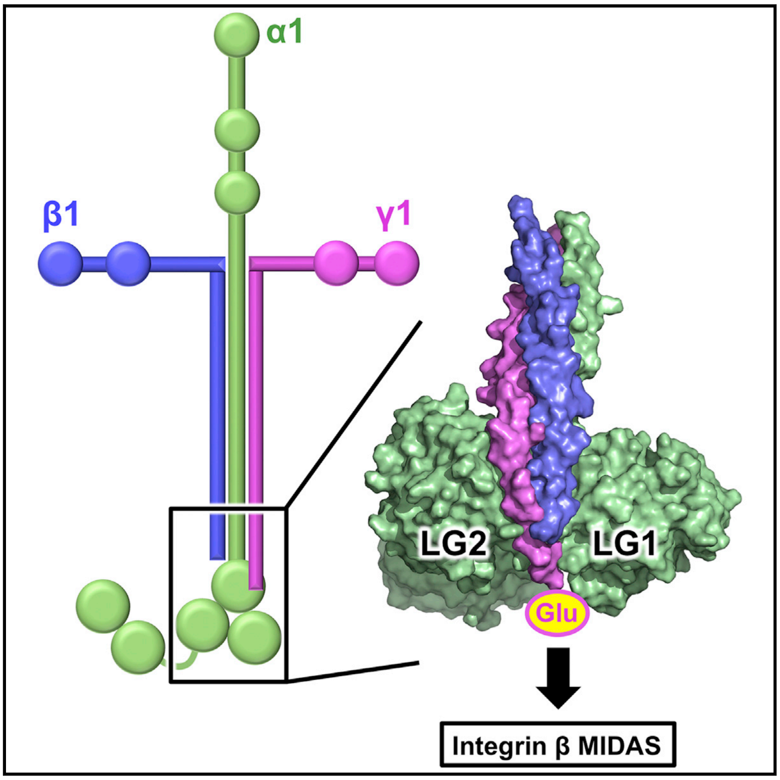

Highlights

- The crystal structure of an integrin-binding fragment of laminin has been determined
- The interactions between the coiled coil and the α chain LG domains are revealed
- The γ1 chain tail is located on the surface of α chain domains LG1 and LG2
- Integrins are proposed to bind directly to a glutamic acid in the γ1 chain tail

Authors

David Pulido, Sadaf-Ahmahni Hussain, Erhard Hohenester

Correspondence

e.hohenester@imperial.ac.uk

In Brief

The heterotrimeric laminins are important cell-adhesive proteins. Pulido et al. have determined the crystal structure of a minimal integrin-binding fragment of laminin-111, which reveals the quaternary interactions essential for integrin binding.

Accession Numbers

5MC9

Pulido et al., 2017, Structure *25*, 530–535
March 7, 2017 © 2017 The Author(s). Published by Elsevier Ltd.
http://dx.doi.org/10.1016/j.str.2017.01.002

CellPress

Structure
Short Article

Crystal Structure of the Heterotrimeric Integrin-Binding Region of Laminin-111

David Pulido,[1] Sadaf-Ahmahni Hussain,[1] and Erhard Hohenester[1,2,*]
[1]Department of Life Sciences, Imperial College London, Sir Ernst Chain Building, London SW7 2AZ, UK
[2]Lead Contact
*Correspondence: e.hohenester@imperial.ac.uk
http://dx.doi.org/10.1016/j.str.2017.01.002

SUMMARY

Laminins are cell-adhesive glycoproteins that are essential for basement membrane assembly and function. Integrins are important laminin receptors, but their binding site on the heterotrimeric laminins is poorly defined structurally. We report the crystal structure at 2.13 Å resolution of a minimal integrin-binding fragment of mouse laminin-111, consisting of ∼50 residues of α1β1γ1 coiled coil and the first three laminin G-like (LG) domains of the α1 chain. The LG domains adopt a triangular arrangement, with the C terminus of the coiled coil situated between LG1 and LG2. The critical integrin-binding glutamic acid residue in the γ1 chain tail is surface exposed and predicted to bind to the metal ion-dependent adhesion site in the integrin β1 subunit. Additional contacts to the integrin are likely to be made by the LG1 and LG2 surfaces adjacent to the γ1 chain tail, which are notably conserved and free of obstructing glycans.

INTRODUCTION

Basement membranes (BMs) are a type of extracellular matrix that underlies all epithelia and surrounds muscle, fat, and peripheral nerve cells (Yurchenco, 2011). BMs are composed of a characteristic set of evolutionarily ancient glycoproteins: laminins, type IV and type XVIII collagens, nidogen, and perlecan (Hynes, 2012). BM assembly begins with the polymerization of laminins at the cell surface; this process requires cellular receptors, including integrins, α-dystroglycan, and sulfated carbohydrates (Hohenester and Yurchenco, 2013). Integrin-mediated cell adhesion to laminins is essential for embryo development and for tissue function in adult animals (Yamada and Sekiguchi, 2015; Yurchenco, 2011). Disruption of the laminin-integrin interaction causes a severe skin-blistering disorder in humans (Has and Nystrom, 2015); the interaction is also perturbed in many cancers (Ramovs et al., 2016). Moreover, integrin-mediated adhesion to laminin supports the long-term self-renewal of stem cells in culture (Miyazaki et al., 2012; Rodin et al., 2010).

Laminins are large heterotrimers composed of one of five α chains, one of three β chains, and one of three γ chains (Aumailley et al., 2005). The laminin-binding integrins α3β1, α6β1, α7β1, and α6β4 have distinct specificities for the 15 known laminin heterotrimers (Nishiuchi et al., 2006). Laminin-111 (α1β1γ1) was originally purified from the Engelbreth-Holm-Swarm (EHS) mouse tumor and has been extensively studied for nearly 40 years. The N-terminal regions of the α1, β1, and γ1 chains form the short arms of the cross-shaped laminin molecule, and their C-terminal regions associate to form a long α-helical coiled coil (Beck et al., 1990). The α1 chain continues for another ∼1,000 residues after the coiled coil, which are folded into five consecutive laminin G-like (LG) domains (Timpl et al., 2000). It has long been known that integrin binding requires the native quaternary structure of laminin: first, a proteolytic laminin-111 fragment termed E8 (∼220 residues of coiled coil and LG1-LG3) was shown to be sufficient for integrin-mediated cell adhesion, but the activity was lost when E8 was dissociated into its constituent chains (Deutzmann et al., 1990). Second, a glutamic acid in the third position from the C terminus of the γ1 chain (Glu1605 in mouse γ1) was shown to be essential for integrin binding to laminin-511, but the isolated γ1 tail was inactive (Ido et al., 2007). A structural explanation for these observations has been difficult to obtain, however. Here, we report the crystal structure of the integrin-binding region of laminin, which reveals that the critical γ1 tail lies on top of the LG1 and LG2 domains of the α chain. We propose that integrins recognize this composite surface on the laminin heterotrimer.

RESULTS

Structure Determination

To obtain a heterotrimeric laminin fragment amenable to crystallization, we shortened the coiled coil of the E8 fragment from ∼220 to ∼50 residues. Our design of this mini-E8 fragment was inspired by a peptide study, which showed that ∼50 residues are sufficient for a stable laminin α2β1γ1 coiled coil (Nomizu et al., 1996). Co-expression of the α1, β1, and γ1 chains of mini-E8 resulted in a stable heterotrimer with the expected disulfide bond between the β1 and γ1 chains (Figures 1A and 1B). HT1080 fibrosarcoma cells, which adhere to laminin-111 and E8 using integrin α6β1 (Brown and Goodman, 1991; Deutzmann et al., 1990), adhered equally well to mini-E8, and cell adhesion was abrogated by mutation of the critical integrin-binding residue, γ1 E1605Q (Figure 1C). The crystal structure of enzymatically deglycosylated mini-E8 was determined to a resolution of 2.13 Å (Table 1). The crystallographic model is complete except for residues 2,539–2,546 in α1 LG3 and the last two residues of γ1.

 © 2017 The Author(s). Published by Elsevier Ltd.
This is an open access article under the CC BY license (http://creativecommons.org/licenses/by/4.0/).

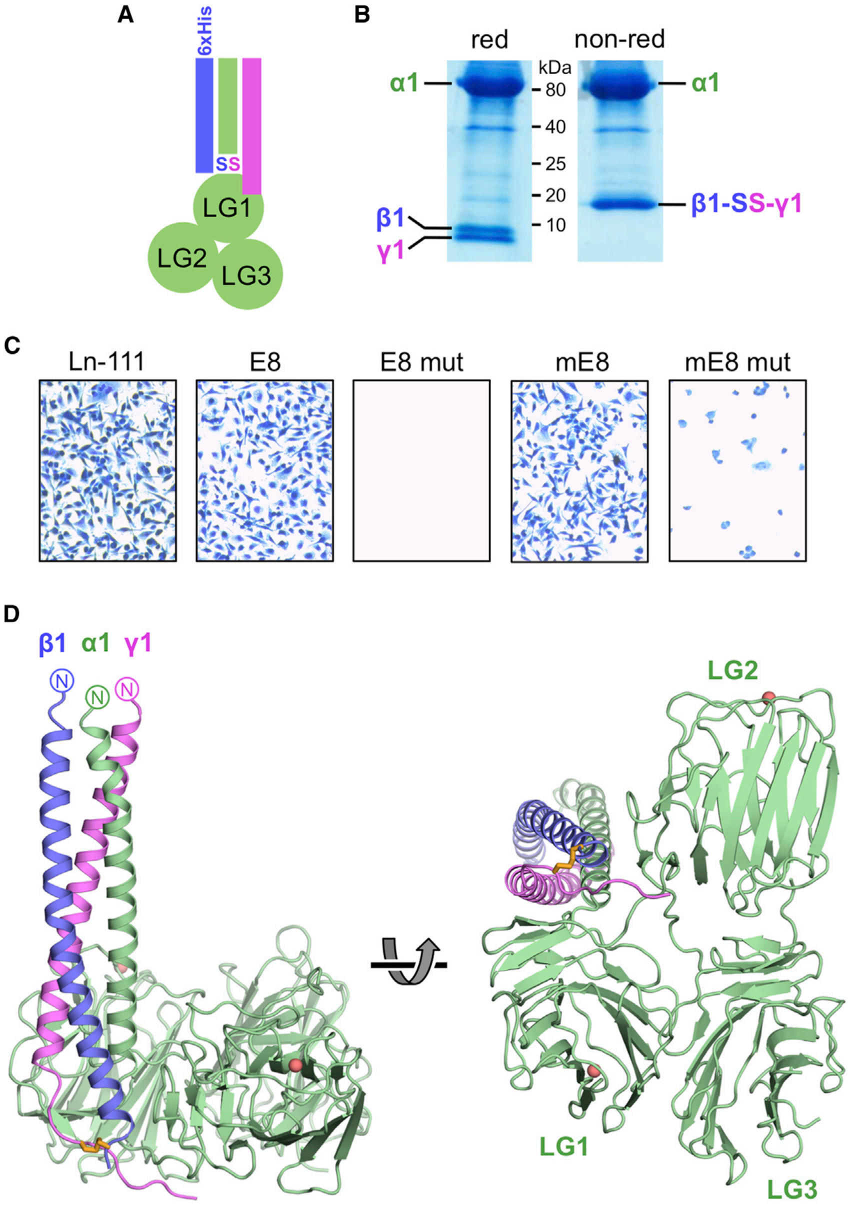

Figure 1. Structure of the Integrin-Binding Region of Laminin-111

(A) Schematic drawing of laminin-111 mini-E8 (α1 chain, green; β1 chain, blue; γ1 chain, magenta). The disulfide bond between β1 and γ1 is indicated.

(B) Reducing and non-reducing SDS-PAGE analysis of mini-E8 pulled down from the 293F cell culture supernatant using Ni^{2+}-affinity resin.

(C) Adhesion of HT1080 fibrosarcoma cells to mouse EHS tumor laminin-111 (Ln-111), recombinant E8, recombinant mini-E8 (mE8), and their respective γ1 E1605Q mutants (mut). Data shown are representative of four independent experiments.

(D) Two orthogonal views of the mini-E8 crystal structure. The disulfide bond linking the C termini of the β1 and γ1 chains is shown in orange. The Ca^{2+} ions in LG1 and LG2 are shown as salmon-colored spheres. The C-terminal residue of the γ1 chain in this structure is Glu1605. See also Figure S1.

Overall Structure of Laminin-111 Mini-E8

The three LG domains of the α1 chain in mini-E8 are arranged in a triangle (Figure 1D), in agreement with negative-stain electron micrographs of laminin-111 (Bruch et al., 1989). The coiled coil of mini-E8 is attached perpendicularly to the LG1-LG3 triangle, with its C terminus located between LG1 and LG2. In a previous crystal structure of the isolated LG1-LG3 domains of the laminin α2 chain, LG1 was completely dissociated from LG2 and LG3 (Carafoli et al., 2009). Thus, the presence of the coiled coil is required to establish the triangular LG arrangement in mini-E8. Of the three helices making up the coiled coil, the α1 helix is closest to the center of the triangle. LG1 interacts extensively with the coiled coil (see below). The LG1-LG2 linker passes near the C terminus of the coiled coil and places LG2 on the opposite side of the coiled coil. The association of LG2 with the coiled coil is remarkably tenuous: apart from a single direct interaction involving α1 Tyr2483, all the contacts are mediated by water molecules. The interface between LG2 and LG3 is also dominated by water-mediated contacts. LG3 is farthest from the coiled coil and closes the triangle by forming an extensive interface with LG1. The β1 and γ1 helices are connected by a disulfide bridge near their C termini. The γ1 chain extends for another seven residues, which fold over the α1 LG1-LG2 linker and position the critical γ1 Glu1605 residue on the surface between the LG1 and LG2 domains (Figure 1).

The mini-E8 structure is in excellent agreement with a recent crosslinking study of laminin-111 (Armony et al., 2016). The authors observed crosslinks from α1 Lys2119 to LG1, LG2, and the LG2-LG3 linker, consistent with the location of Lys2119 in the penultimate turn of the α1 helix and its side chain pointing toward the center of the LG1-LG3 triangle (Figure S1A). Furthermore, crosslinks were observed from γ1 Lys1606 (adjacent to the critical γ1 Glu1605) to LG1 and LG2, consistent with the position of the γ1 tail in our structure (Figure S1B).

The Heterotrimeric Coiled Coil of Mini-E8

The coiled coil in mini-E8 spans seven heptad repeats, the first two of which have weak electron density. The heptads adopt the standard arrangement (Cohen and Parry, 1990), with predominantly aliphatic residues in the *a* and *d* positions forming the core of the helical bundle (Figure 2). Unusually for a coiled coil, the α1 helix is completely straight, except for a kink at Pro2095. When the coiled coil is viewed from its C terminus, α1, β1, and γ1 are arranged in a counterclockwise order. Our mini-E8 structure thus confirms the register and chain order proposed in the recent crosslinking study (Armony et al., 2016). Sequence comparison shows that the very C terminus of

Table 1. Crystallographic Statistics

Data Collection	
Wavelength (Å)	0.920
Resolution range (Å)	56.9–2.13 (2.19–2.13)
Space group	$P2_12_12_1$
Unit cell dimensions	
a, *b*, *c* (Å)	62.76, 98.36, 135.24
α, β, γ (°)	90, 90, 90
Unique reflections	47,309
Multiplicity	4.3 (4.3)
Completeness (%)	99.4 (99.8)
Mean I/σ(I)	11.5 (1.9)
$CC_{1/2}$	0.997 (0.628)
R_{merge}	0.092 (0.780)
Refinement	
Protein atoms	5,330
Solvent atoms	294 H_2O, 2 Ca^{2+}
R_{work}	0.210
R_{free}	0.238
RMSD bonds (Å)	0.004
RMSD angles (°)	0.70
Ramachandran plot	
Favored (%)	97.1
Allowed (%)	2.9
Outliers (%)	0

RMSD, root-mean-square deviation.

the coiled coil is conserved in all laminin isoforms (Figure 2). However, the α4 and α5 chains deviate from the regular heptad pattern in the N-terminal half of the mini-E8 coiled coil, and may require additional regions for stable association with the β and γ chains.

Interactions Involving the C Terminus of the Coiled Coil

The β1 helix of the coiled coil is continuous all the way to the C-terminal β1-γ1 disulfide bond. In contrast, the α1 and γ1 helices terminate two turns earlier, and their C-terminal regions interact with each other and with LG1 (Figure 3A). The α1 chain straightens after Ile2124 to become the first β strand of LG1 at Ala2127. The γ1 chain bulges after Pro1595, with the side chain of Phe1599 pointing back toward Pro1595. The opening of the coiled coil near its C terminus creates a hydrophobic pocket that accommodates the α1 LG1 residue Trp2293, thereby establishing the intimate interface between LG1 and the coiled coil (Figure 3A). Sequence comparison suggests that similar quaternary interactions are formed in all laminin heterotrimers: aromatic residues corresponding to α1 Trp2293 and Tyr2295 are present in all other α chains (Carafoli et al., 2009), and a proline corresponding to γ1 Pro1595 is present in all γ chains (Figure 2).

The γ1 tail, which is critical for integrin binding (Ido et al., 2007), is almost entirely resolved in our mini-E8 structure; only the Glu1605 side chain and the C-terminal two residues are missing (Figure 3B). The side chain of γ1 Asn1600 (conserved in all vertebrate γ1 sequences) forms two hydrogen bonds with the two C-terminal peptide carbonyl groups of the β1 helix. Further along, the γ1 tail forms two main-chain hydrogen bonds with the α1 LG1-LG2 linker. Thus, a complex network of quaternary interactions organizes the C-terminal region of the coiled coil, including the γ1 tail. We note that the LG1 and LG2 surfaces on either side of the γ1 tail are more highly conserved than LG3, and that none of the five N-linked glycosylation sites of mini-E8 are located in this region (Figure 3C).

The LG1-LG3 Interface

The LG1-LG3 interface is the largest interdomain contact in mini-E8 (Figure S2), yet the interface residues are not conserved in other laminin α chains. For instance, α1 LG3 residue Ala2661, which points into a snug pocket on the LG1 surface, is replaced by arginine in the α3, α4, and α5 chains (Carafoli et al., 2009). The lack of conservation in this region sharply contrasts the strict conservation of quaternary interactions near the C terminus of the coiled coil, and suggests that the precise nature of the LG1-LG3 interface may be less important, as long as a circular LG1-LG2-LG3 structure is established.

DISCUSSION

The broad features of the quaternary structure of laminins were established many years ago (Beck et al., 1990). Since then, crystal structures have been determined of several key regions (Carafoli et al., 2012; Hohenester and Yurchenco, 2013; Moran et al., 2015; Pulido et al., 2016), but the coiled coil and the integrin-binding region have defied all crystallographic attempts to date. Our high-resolution crystal structure of laminin-111 mini-E8 now reveals how the coiled coil is integrated into the LG tandem, and provides a satisfying explanation as to why no single laminin chain is sufficient for integrin binding (Yamada and Sekiguchi, 2015).

The identification of γ1 Glu1605 as a key residue for integrin α6β1 binding suggested two possible scenarios: either this residue binds directly to the integrin, or it is required to organize the LG tandem into a competent conformation (Ido et al., 2007). Our structure strongly favors the first scenario. Because the γ1 tail is located on the surface and appears to be quite mobile, it is difficult to imagine how γ1 Glu1605 could be essential for quaternary structure. It is more likely that this acidic residue is the ligand for the metal ion-dependent adhesion site in the integrin β1 subunit, similarly to the aspartic acid of the famous Arg-Gly-Asp motif in the Fn10 domain of fibronectin (Xiong et al., 2002). This interpretation is in line with results from exhaustive mutagenesis of the laminin α5 chain, which failed to identify acidic residues within the LG1-LG3 region that are essential for integrin binding (Yamada and Sekiguchi, 2015). In fibronectin, the so-called synergy site in the Fn9 domain makes additional contacts to the integrin α subunit (Nagae et al., 2012). In laminin-111, an equivalent function may be provided by the conserved LG1 and LG2 surfaces adjacent to the γ1 tail. This interpretation would be consistent with the finding that the integrin specificity of laminin heterotrimers is largely determined by the α chain (Nishiuchi et al., 2006). It is also noteworthy that the epitopes of two function-blocking antibodies map to the LG1 and LG2 domains, respectively (Ido et al., 2006; Yamashita et al., 2010). Because no structure of a laminin-binding integrin is available, and

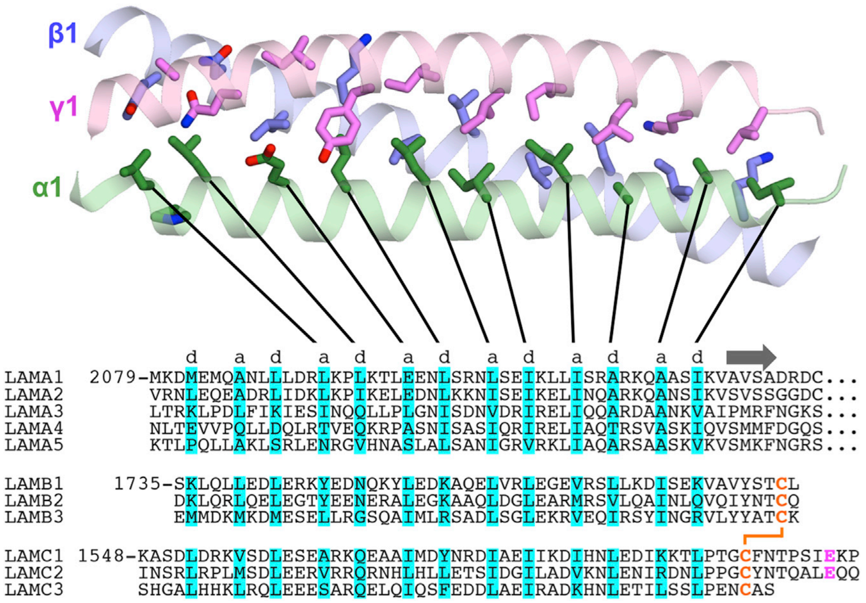

Figure 2. Structure and Sequence Conservation of the Coiled Coil

The N termini of the three helices (α1 chain, green; β1 chain, blue; γ1 chain, magenta) are on the left. The side chains of residues in the *a* and *d* positions of the heptad repeats (Cohen and Parry, 1990) are shown as sticks. Below the structure is an alignment of all mouse laminin chains with the *a* and *d* positions highlighted in cyan. The first β strand of the α1 LG1 domain is indicated by an arrow. The β1-γ1 inter-chain disulfide bond and the critical glutamic acid in the γ1 chain are in orange and magenta, respectively.

because the laminin γ1 tail could reorient substantially upon integrin binding, it is difficult to predict how the LG1 or LG2 domains might contact the integrin. The requirement of LG3 for function is likely to be indirect: removal of LG3 (Ido et al., 2004) would destabilize the quaternary structure, and replacement with LG3 from another α chain (Kikkawa et al., 2007) would not be tolerated because of the lack of conservation in the LG1-LG3 interface.

In summary, our structure of the integrin-binding region of laminin-111 rationalizes a large number of biochemical findings and provides the framework for future studies into the mechanism of laminin recognition by integrins.

EXPERIMENTAL PROCEDURES

Expression Vectors

All coding sequences were obtained by PCR amplification from cDNAs kindly provided by Peter Yurchenco. Our numbering scheme is based on the UniProt entries for mouse laminin α1 (UniProt: P19137), mouse laminin β1 (UniProt: P02469), and mouse laminin γ1 (UniProt: P02468). The E8 fragment spans α1 residues 1,911–2,707, β1 residues 1,561–1,786, and γ1 residues 1,362–1,607. The mini-E8 fragment spans α1 residues 2,079–2,707, β1 residues 1,735–1,786, and γ1 residues 1,548–1,607. The inserts were cloned into modified pCEP vectors (Kohfeldt et al., 1997). The α1 and γ1 constructs were made without tags and contain a vector-derived APLA sequence at their N terminus. The β1 constructs for biochemical experiments were made with a fused N-terminal His-tag (APLVHHHHHHALA). The β1 construct for crystallization was made with a tobacco etch virus (TEV) protease-cleavable His-tag (Pulido et al., 2016), leaving an N-terminal GALA sequence after TEV protease treatment. The γ1 E1605Q mutants of E8 and mini-E8 were made using QuikChange II XL (Agilent Technologies).

Protein Expression and Purification

The heterotrimeric E8 and mini-E8 fragments, as well as their γ1 E1605Q mutants, were produced using the FreeStyle 293 Expression System (Thermo Fisher Scientific) following the manufacturer's protocols. In brief, 293F cells were grown in a shaking incubator at 37°C with 8% CO_2 in serum-free FreeStyle 293 Expression Medium to a cell density of 10^6 cells/mL. The cells were cotransfected with the respective expression vectors at a 1:1:1 molar ratio. Transfections were performed using polyethylenimine (PEI; VWR International) and a DNA:PEI ratio of 1:3 (w/w). The conditioned media containing the secreted heterotrimers were collected 72 hr after transfection. The filtered media were adjusted to a final concentration of 20 mM Na-HEPES (pH 7.5) and loaded onto 5-mL HisTrap excel columns (GE Healthcare) using an Äkta pure chromatography system (GE Healthcare). The columns were washed with 20 mM Na-HEPES and 150 mM NaCl (pH 7.5), and the proteins were eluted with the same buffer containing 200 mM imidazole. Fractions containing protein were concentrated using Vivaspin centrifugal devices (Sartorius) to a final concentration of 1 mg/mL and further purified on a Superdex 200 10/300 column (GE Healthcare) using 20 mM Tris-HCl and 150 mM NaCl (pH 7.5) as the running buffer.

The mini-E8 protein for crystallization was produced as described above using the FreeStyle 293 Expression System, but with 5 μM kifunensine (Industrial Research) added to the growth medium. Twenty-four hours after transfection the conditioned medium was collected, and the transfected cells were resuspended in fresh kifunensine-containing medium, and further incubated for 48 hr. The protein was purified from the combined conditioned media using HisTrap affinity chromatography and concentrated to 1 mg/mL. This protein solution was then dialyzed into 20 mM Tris-HCl and 150 mM NaCl (pH 7.5) and simultaneously digested with His-tagged TEV protease and His-tagged EndoH endoglycosidase (made in *E. coli* using expression vectors kindly provided by Stephen Curry and Daniel Leahy, respectively) at enzyme:substrate ratios of 1:10 for 18 hr at room temperature. The reaction mixture was loaded onto a 1-mL HisTrap FF column (GE Healthcare) and the flowthrough collected. The final purification step was done by size-exclusion chromatography, as described above.

Cell Adhesion Assay

Cell adhesion assays were performed using HT1080 human fibrosarcoma cells (ATCC). The cells were grown at 37°C with 5% CO_2 in DMEM containing 10% fetal bovine serum. Twenty-four-well plates (Corning Life Sciences) were coated with 100 nM mouse EHS tumor laminin-111 (Sigma-Aldrich), recombinant E8, recombinant mini-E8, or the respective γ1 E1605Q mutants, at 4°C overnight. The following day, the wells were blocked with 2% BSA in PBS for 2 hr at room temperature. HT1080 cells were harvested, centrifuged, and resuspended in serum-free DMEM at a density of 3 × 10^5 cells/mL, and then plated on the coated wells. After incubation at 37°C for 30 min, the attached cells were fixed, stained with Diff-Quik (International Reagents), and photographed using an inverted microscope.

Reductive Methylation

Deglycosylated mini-E8 was reductively methylated using the Reductive Alkylation Kit (Hampton Research) following the manufacturer's protocol. In brief, 1 mL of a 5 mg/mL solution of mini-E8 was reacted with dimethylamine borane complex solution and formaldehyde at 4°C for 22 hr. The reaction was stopped by addition of glycine. The reaction mixture was concentrated using a Vivaspin device and further purified by size-exclusion chromatography, as described above.

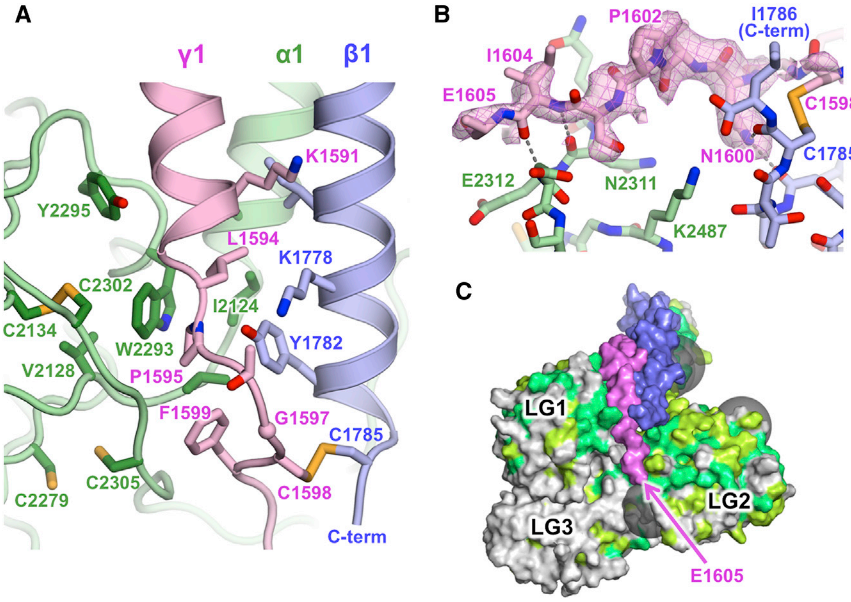

Figure 3. Quaternary Interactions near the C terminus of the Coiled Coil

(A) Interactions of α1 LG1 (green) with the C-terminal regions of the β1 (blue) and γ1 (pink) chains. The Cys2279-Cys2305 disulfide bond is reduced due to radiation damage.

(B) Interactions of the γ1 tail (pink) with the α1 LG1-LG2 linker (green) and the C terminus of the β1 chain (blue). A simulated annealing omit map of the γ1 tail is shown as a pink mesh ($F_{obs} - F_{calc}$, 2σ contouring). There is no electron density for the side chain of γ1 Glu1605.

(C) Surface conservation of the α1 chain (green, strictly conserved in 14 vertebrate laminin α1 sequences; yellow-green, strongly similar). The positions of N-linked glycans are indicated by transparent gray spheres. See also Figure S2.

Crystallization

Screening was done at 20°C by the sitting-drop vapor diffusion method using 96-well plates (Greiner) and a range of commercial screens. A mosquito Nanolitre Robot (TTP Labtech) was used to set up 200 nL drops. The initial crystals of deglycosylated and methylated mini-E8 were obtained in the JCSG+ screen (Molecular Dimensions) using a protein concentration of 17 mg/mL and 0.02 M magnesium chloride hexahydrate, 0.1 M Na-HEPES (pH 7.5), and 22% (w/v) poly(acrylic acid sodium salt) 5,100 as precipitant. Larger crystals were grown in 2 μL hanging drops using the same precipitant solution. After 2 weeks, the crystals were flash-frozen in liquid nitrogen using reservoir solution supplemented with 20% ethylene glycol as cryoprotectant.

Crystal Structure Determination

Diffraction data were collected at 100 K at beamline I04-1 of the Diamond Light Source (Oxfordshire, UK). The data were processed using XDS (Kabsch, 2010) and programs of the CCP4 suite (Winn et al., 2011) as implemented in the xia2 pipeline (Winter et al., 2013). $CC_{1/2}$ was used to determine the resolution limit (Karplus and Diederichs, 2012). The phases were determined by molecular replacement using PHASER as implemented in the PHENIX suite (Adams et al., 2010). The search models were derived from the crystal structure of the LG1-LG3 region of the laminin α2 chain (Carafoli et al., 2009). Manual rebuilding and refinement were done using COOT (Emsley and Cowtan, 2004) and PHENIX. The figures were generated using PyMOL (www.pymol.org).

Sequence Alignment

For the analysis of surface conservation in Figure 3C, we aligned the laminin α1 sequences of 14 vertebrate species using Clustal Omega (Sievers et al., 2011): human (UniProt: 25391), mouse (UniProt: P19137), rat (UniProt: D4A409), sheep (UniProt: W5NQ90), cow (UniProt: F1MEG3), dog (UniProt: F1PJ02), cat (UniProt: M3W9Z2), panda (UniProt: G1LM02), rabbit (UniProt: G1TTH9), chick (UniProt: F1NM47), bat (UniProt: G1P1G9), zebrafish (UniProt: Q45H72), *Xenopus tropicalis* (UniProt: F7AVS9), and *Oreochromis niloticus* (UniProt: I3KD18). Residues flagged by a colon (:) in the alignment were considered to be strongly similar.

ACCESSION NUMBERS

The coordinates of the laminin-111 mini-E8 structure have been deposited in the PDB under accesion code PDB: 5MC9.

SUPPLEMENTAL INFORMATION

Supplemental Information includes two figures and can be found with this article online at http://dx.doi.org/10.1016/j.str.2017.01.002.

AUTHOR CONTRIBUTIONS

All authors contributed to experimental design and data analysis. D.P. produced and purified the proteins, carried out the cell adhesion experiments, and determined the crystal structure. S.H. assisted with protein production. E.H. designed and supervised the study. D.P. and E.H. wrote the manuscript.

ACKNOWLEDGMENTS

We acknowledge Diamond Light Source for time on beamline I04-1 under proposal MX9424. This work was funded by a Wellcome Trust Senior Investigator Award to E.H. (101748/Z/13/Z).

Received: November 19, 2016
Revised: December 16, 2016
Accepted: January 5, 2017
Published: January 26, 2017

REFERENCES

Adams, P.D., Afonine, P.V., Bunkoczi, G., Chen, V.B., Davis, I.W., Echols, N., Headd, J.J., Hung, L.W., Kapral, G.J., Grosse-Kunstleve, R.W., et al. (2010). PHENIX: a comprehensive Python-based system for macromolecular structure solution. Acta Crystallogr. D Biol. Crystallogr. *66*, 213–221.

Armony, G., Jacob, E., Moran, T., Levin, Y., Mehlman, T., Levy, Y., and Fass, D. (2016). Cross-linking reveals laminin coiled-coil architecture. Proc. Natl. Acad. Sci. USA *113*, 13384–13389.

Aumailley, M., Bruckner-Tuderman, L., Carter, W.G., Deutzmann, R., Edgar, D., Ekblom, P., Engel, J., Engvall, E., Hohenester, E., Jones, J.C., et al. (2005). A simplified laminin nomenclature. Matrix Biol. *24*, 326–332.

Beck, K., Hunter, I., and Engel, J. (1990). Structure and function of laminin: anatomy of a multidomain glycoprotein. FASEB J. *4*, 148–160.

Brown, J.C., and Goodman, S.L. (1991). Different cellular receptors for human placental laminin and murine EHS laminin. FEBS Lett. *282*, 5–8.

Bruch, M., Landwehr, R., and Engel, J. (1989). Dissection of laminin by cathepsin G into its long-arm and short-arm structures and localization of

regions involved in calcium dependent stabilization and self-association. Eur. J. Biochem. *185*, 271–279.

Carafoli, F., Clout, N.J., and Hohenester, E. (2009). Crystal structure of the LG1-3 region of the laminin α2 chain. J. Biol. Chem. *284*, 22786–22792.

Carafoli, F., Hussain, S.A., and Hohenester, E. (2012). Crystal structures of the network-forming short-arm tips of the laminin β1 and γ1 chains. PLoS One *7*, e42473.

Cohen, C., and Parry, D.A. (1990). α-Helical coiled coils and bundles: how to design an α-helical protein. Proteins *7*, 1–15.

Deutzmann, R., Aumailley, M., Wiedemann, H., Pysny, W., Timpl, R., and Edgar, D. (1990). Cell adhesion, spreading and neurite stimulation by laminin fragment E8 depends on maintenance of secondary and tertiary structure in its rod and globular domain. Eur. J. Biochem. *191*, 513–522.

Emsley, P., and Cowtan, K. (2004). Coot: model-building tools for molecular graphics. Acta Crystallogr. D Biol. Crystallogr. *60*, 2126–2132.

Has, C., and Nystrom, A. (2015). Epidermal basement membrane in health and disease. Curr. Top. Membr. *76*, 117–170.

Hohenester, E., and Yurchenco, P.D. (2013). Laminins in basement membrane assembly. Cell Adh. Migr. *7*, 56–63.

Hynes, R.O. (2012). The evolution of metazoan extracellular matrix. J. Cell Biol. *196*, 671–679.

Ido, H., Harada, K., Futaki, S., Hayashi, Y., Nishiuchi, R., Natsuka, Y., Li, S., Wada, Y., Combs, A.C., Ervasti, J.M., et al. (2004). Molecular dissection of the α-dystroglycan- and integrin-binding sites within the globular domain of human laminin-10. J. Biol. Chem. *279*, 10946–10954.

Ido, H., Harada, K., Yagi, Y., and Sekiguchi, K. (2006). Probing the integrin-binding site within the globular domain of laminin-511 with the function-blocking monoclonal antibody 4C7. Matrix Biol. *25*, 112–117.

Ido, H., Nakamura, A., Kobayashi, R., Ito, S., Li, S., Futaki, S., and Sekiguchi, K. (2007). The requirement of the glutamic acid residue at the third position from the carboxyl termini of the laminin γ chains in integrin binding by laminins. J. Biol. Chem. *282*, 11144–11154.

Kabsch, W. (2010). Xds. Acta Crystallogr. D Biol. Crystallogr. *66*, 125–132.

Karplus, P.A., and Diederichs, K. (2012). Linking crystallographic model and data quality. Science *336*, 1030–1033.

Kikkawa, Y., Sasaki, T., Nguyen, M.T., Nomizu, M., Mitaka, T., and Miner, J.H. (2007). The LG1-3 tandem of laminin α5 harbors the binding sites of Lutheran/basal cell adhesion molecule and α3β1/α6β1 integrins. J. Biol. Chem. *282*, 14853–14860.

Kohfeldt, E., Maurer, P., Vannahme, C., and Timpl, R. (1997). Properties of the extracellular calcium binding module of the proteoglycan testican. FEBS Lett. *414*, 557–561.

Miyazaki, T., Futaki, S., Suemori, H., Taniguchi, Y., Yamada, M., Kawasaki, M., Hayashi, M., Kumagai, H., Nakatsuji, N., Sekiguchi, K., et al. (2012). Laminin E8 fragments support efficient adhesion and expansion of dissociated human pluripotent stem cells. Nat. Commun. *3*, 1236.

Moran, T., Gat, Y., and Fass, D. (2015). Laminin L4 domain structure resembles adhesion modules in ephrin receptor and other transmembrane glycoproteins. FEBS J. *282*, 2746–2757.

Nagae, M., Re, S., Mihara, E., Nogi, T., Sugita, Y., and Takagi, J. (2012). Crystal structure of α5β1 integrin ectodomain: atomic details of the fibronectin receptor. J. Cell Biol. *197*, 131–140.

Nishiuchi, R., Takagi, J., Hayashi, M., Ido, H., Yagi, Y., Sanzen, N., Tsuji, T., Yamada, M., and Sekiguchi, K. (2006). Ligand-binding specificities of laminin-binding integrins: a comprehensive survey of laminin-integrin interactions using recombinant α3β1, α6β1, α7β1 and α6β4 integrins. Matrix Biol. *25*, 189–197.

Nomizu, M., Utani, A., Beck, K., Otaka, A., Roller, P.P., and Yamada, Y. (1996). Mechanism of laminin chain assembly into a triple-stranded coiled-coil structure. Biochemistry *35*, 2885–2893.

Pulido, D., Briggs, D.C., Hua, J., and Hohenester, E. (2016). Crystallographic analysis of the laminin β2 short arm reveals how the LF domain is inserted into a regular array of LE domains. Matrix Biol. http://dx.doi.org/10.1016/j.matbio.2016.06.006.

Ramovs, V., Te Molder, L., and Sonnenberg, A. (2016). The opposing roles of laminin-binding integrins in cancer. Matrix Biol. http://dx.doi.org/10.1016/j.matbio.2016.08.007.

Rodin, S., Domogatskaya, A., Strom, S., Hansson, E.M., Chien, K.R., Inzunza, J., Hovatta, O., and Tryggvason, K. (2010). Long-term self-renewal of human pluripotent stem cells on human recombinant laminin-511. Nat. Biotechnol. *28*, 611–615.

Sievers, F., Wilm, A., Dineen, D., Gibson, T.J., Karplus, K., Li, W., Lopez, R., McWilliam, H., Remmert, M., Soding, J., et al. (2011). Fast, scalable generation of high-quality protein multiple sequence alignments using Clustal Omega. Mol. Syst. Biol. *7*, 539.

Timpl, R., Tisi, D., Talts, J.F., Andac, Z., Sasaki, T., and Hohenester, E. (2000). Structure and function of laminin LG modules. Matrix Biol. *19*, 309–317.

Winn, M.D., Ballard, C.C., Cowtan, K.D., Dodson, E.J., Emsley, P., Evans, P.R., Keegan, R.M., Krissinel, E.B., Leslie, A.G., McCoy, A., et al. (2011). Overview of the CCP4 suite and current developments. Acta Crystallogr. D Biol. Crystallogr. *67*, 235–242.

Winter, G., Lobley, C.M., and Prince, S.M. (2013). Decision making in xia2. Acta Crystallogr. D Biol. Crystallogr. *69*, 1260–1273.

Xiong, J.P., Stehle, T., Zhang, R., Joachimiak, A., Frech, M., Goodman, S.L., and Arnaout, M.A. (2002). Crystal structure of the extracellular segment of integrin αVβ3 in complex with an Arg-Gly-Asp ligand. Science *296*, 151–155.

Yamada, M., and Sekiguchi, K. (2015). Molecular basis of laminin-integrin interactions. Curr. Top. Membr. *76*, 197–229.

Yamashita, H., Shang, M., Tripathi, M., Jourquin, J., Georgescu, W., Liu, S., Weidow, B., and Quaranta, V. (2010). Epitope mapping of function-blocking monoclonal antibody CM6 suggests a "weak" integrin binding site on the laminin-332 LG2 domain. J. Cell Physiol. *223*, 541–548.

Yurchenco, P.D. (2011). Basement membranes: cell scaffoldings and signaling platforms. Cold Spring Harb. Perspect. Biol. *3*, a004911.

Structure, Volume *25*

Supplemental Information

Crystal Structure of the Heterotrimeric Integrin-Binding Region of Laminin-111

David Pulido, Sadaf-Ahmahni Hussain, and Erhard Hohenester

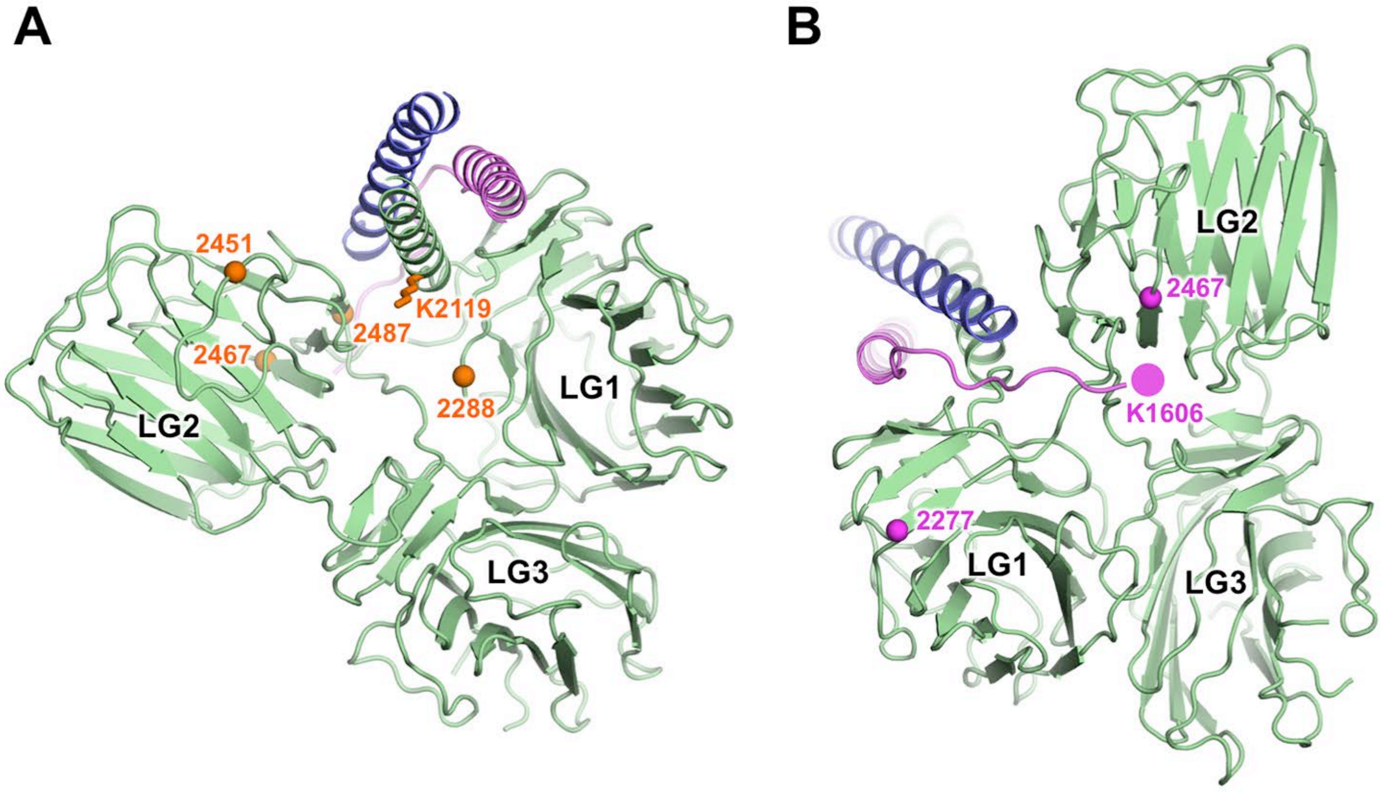

Figure S1, related to Figure 1. Mapping of chemical cross-links onto the mini-E8 structure. The cross-link data are from Armony et al. (2016). (**A**) Cross-links within the laminin α1 chain. α1 Lys2119 (orange sticks) forms cross-links with α1 Lys2288, α1 Lys2451, α1 Lys2467 and α1 Lys2486 (orange Cα spheres). (**B**) Cross-links between the laminin γ1 tail and the α1 chain. γ1 Lys1606 (magenta filled circle adjacent to the last resolved residue of γ1 chain, Glu1605) forms cross-links with α1 Lys2277 and α1 Lys2467 (magenta spheres).

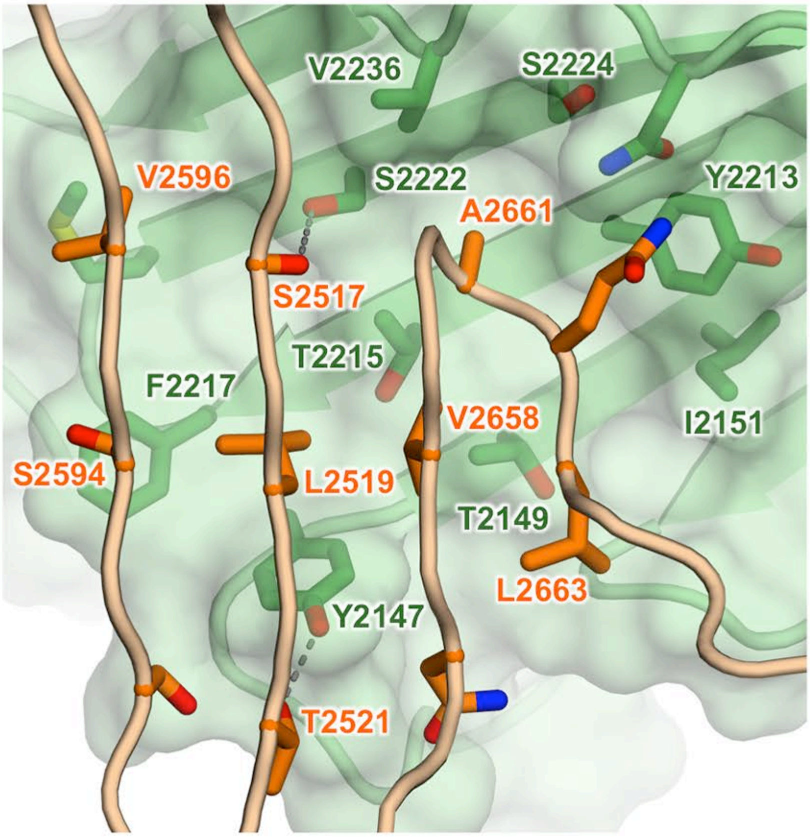

Figure S2, related to Figure 3. The LG1-LG3 interface in laminin-111 mini-E8.
LG1 is shown as a transparent green surface, and LG3 is shown in orange.
